# A Large Retroperitoneal Ganglioneuroma Presenting With an Abdominal Pain: A Case Report

**DOI:** 10.7759/cureus.20600

**Published:** 2021-12-22

**Authors:** Sultanah Bin Gheshayan, Danah Alsadun, Alanood Alharbi, Nahar A Alselaim

**Affiliations:** 1 General Surgery, King Abdulaziz Medical City Riyadh, Riyadh, SAU; 2 General Surgery, King Abdullah International Medical Research Center, Riyadh, SAU; 3 General Surgery, King Saud Bin Abdulaziz University for Health Sciences College of Medicine, Riyadh, SAU; 4 General Surgery, King Abdulaziz Medical City, Riyadh, SAU

**Keywords:** neural crest tumor, neurogenic tumor, lower abdominal pain, retroperitoneal mass, ganglioneuroma

## Abstract

Ganglioneuromas are rare benign neoplasms arising from neural crest tissue. They are a subtype of neurogenic tumors with ganglion cell origin. They are most commonly found in the retroperitoneum and posterior mediastinum. Most ganglioneuromas are found incidentally; most patients are asymptomatic, and it rarely causes symptoms, which are often induced by compression. Here we present a case of a 24-year-old lady, who was investigated for right lower abdominal pain and found to have a right retroperitoneal solid mass at the level of L5-S1, which was displacing the major vessels. The patient underwent open excision of the mass.

## Introduction

Neurogenic tumors are tumors arising from the nervous system. They originate either from the paraganglion system, including pheochromocytomas and paragangliomas, or from the nerve sheath, including neurilemmomas, neurofibromas neurofibromatosis, and malignant nerve sheath tumors [1].

Ganglioneuromas are rare benign neoplasms arising from neural crest tissue [2]. They are subtypes of neurogenic tumors with ganglion cell origin, including ganglioneuromas, ganglioneuroblastomas, and neuroblastomas.

Pathologically, they are characterized by mature sympathetic ganglion cells and Schwann cells in a fibrous stroma and embedded within large cells with abundant cytoplasm [3] Radiologically, they appear as homogeneous well-circumscribed masses on unenhanced computed tomography (CT) [1]. The two most common origins for ganglioneuromas are the retroperitoneum (32%-52% of cases), posterior mediastinum (39%-43% of cases) [1], or cervical region (8%-9% of cases) [4]. Other sites that are rare include the GI tract, parapharyngeal area, and bones [3]. They are mostly found in females, with an approximately 3:2 female/male ratio [5], with an incidence of one per million in a population [5]. They are mostly sporadic, but there are a few cases of ganglioneuromas associated with neurofibromatosis type II and multiple endocrinologic neoplasia type II [5].

Most ganglioneuromas are found incidentally; most patients are asymptomatic and rarely symptomatic, which is often induced by compression [6]. Moreover, some ganglioneuromas can be functional and release catecholaminergic peptides, which can produce hypertensive crises during the surgery [5,6]. They are considered the most benign type of neuroblastic tumors and are treated by complete surgical removal as they may cause compression of adjacent structures and catecholamine release [3]. We present a case of a 24-year-old lady with symptomatic retroperitoneal ganglioneuroma.

## Case presentation

A 24-year-old female was referred from a gynecology clinic with ultrasound findings of a large pelvic mass. The patient presented with a five-year history of chronic right lower abdominal pain, which was vague in nature and associated with her menstrual period. Her past medical, surgical, and family history was insignificant. Physical examination and laboratory work-up were unremarkable.

**Figure 1:**
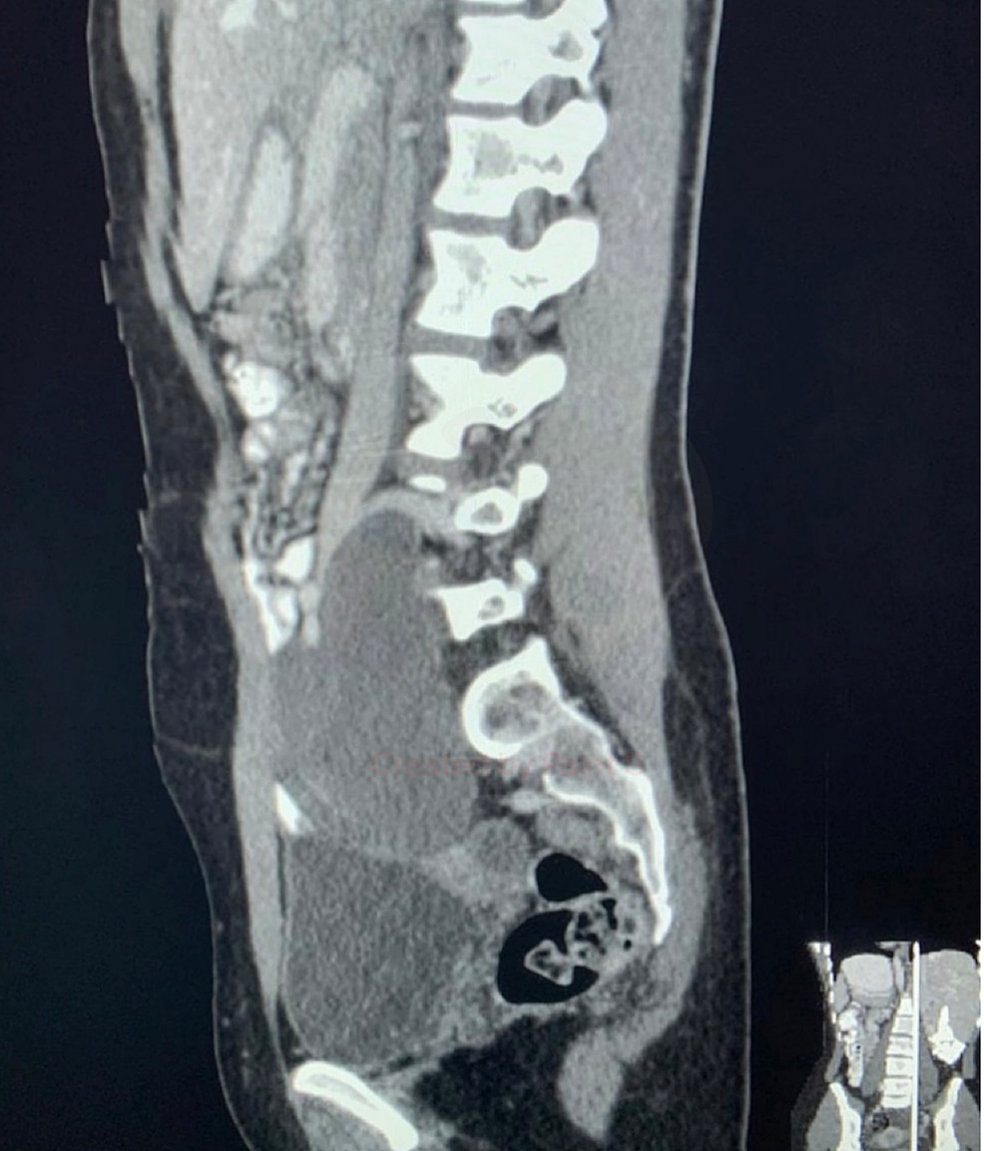
CT image (sagittal view) showing the retroperitoneal mass displacing the inferior vena cava.

CT of the abdomen was performed, which showed an 11.7 x 7 cm right-sided retroperitoneal solid mass at the level of L5-S1; it was displacing the inferior vena cava, the right iliac vessel, and right ureter anteriorly without definite invasion (Figure 1). The mass was homogeneous without calcification, fat, or necrosis, and it showed heterogeneous progressive enhancement on the post-contrast images. The mass was abutting the sacral bone without remodeling or erosion, and the right ureter was displaced anteriorly, with no hydronephrosis. Differential diagnosis included fibrous tumor, retroperitoneal sarcoma, and neurogenic tumor.

**Figure 2:**
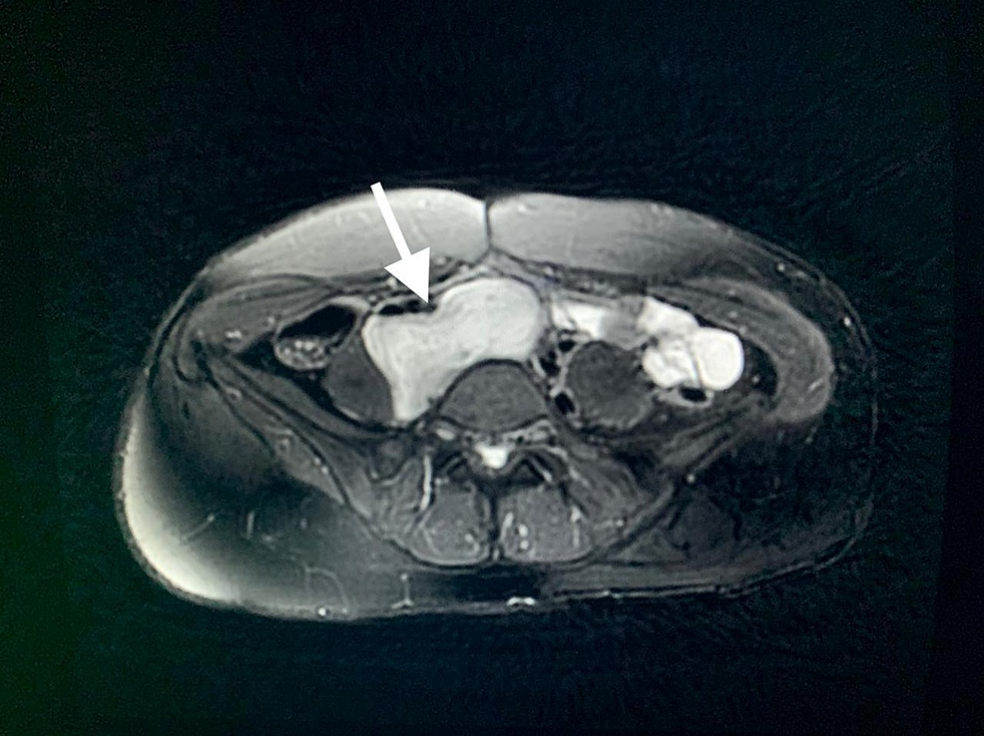
Abdominal MRI T2 featuring the high T2 with streaks of low T2 signal of the mass (pointed with a white arrow).

MRI of the upper abdomen with IV gadolinium was performed; the lesion showed high T2 with streaks of low T2 signal (Figure 2). There was low T1 signal intensity with peripheral enhancement on the early images and faint internal areas of enhancement on delayed images. There was remodeling of the adjacent lumbar and sacral vertebrae with venous collaterals, which suggested a longstanding process.

The case was discussed in the tumor board meeting, and the images were reviewed. The most likely diagnosis was a neurogenic tumor (ganglioglioma), and other differential diagnoses were fibrous tumor and retroperitoneal sarcoma.

The option of CT-guided biopsy was discussed; however, given the difficult access and the lower probability of it to change the management, surgical resection was preferred.

After discussing the treatment options with the patient, the patient agreed to proceed with exploratory laparotomy and retroperitoneal mass excision.

**Figure 3:**
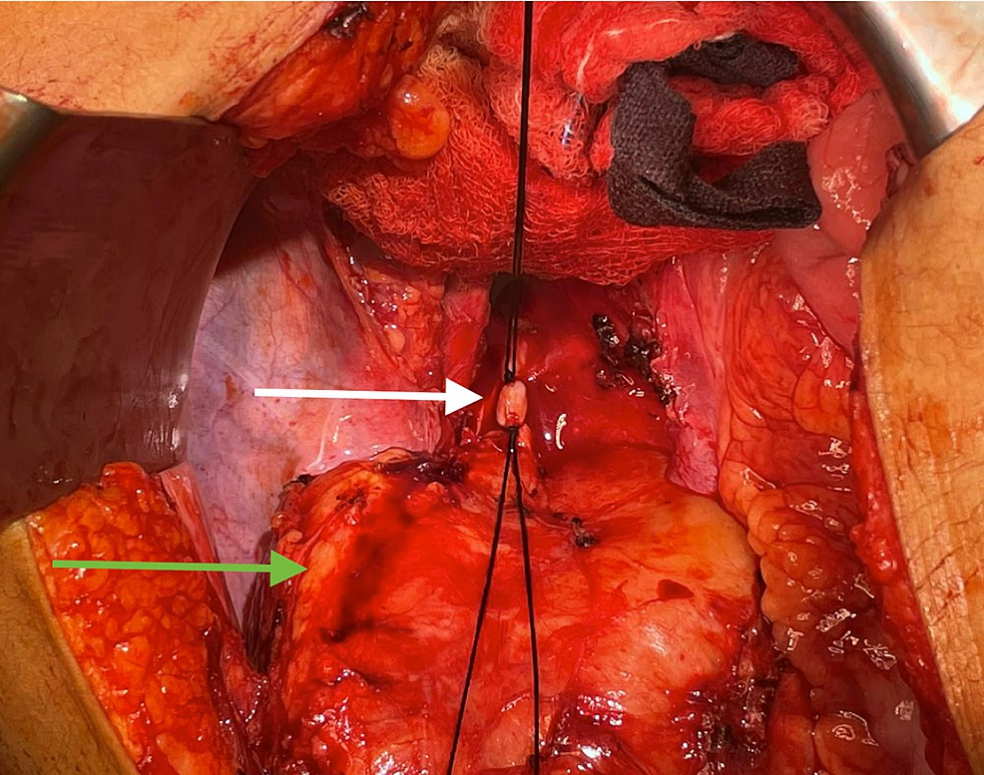
Intraoperative view after completely dissecting the mass and ligating the feeding vessel. The green arrow points to the mass, and the white arrow points to the feeding vessel.

Vascular and urology teams were consulted preoperatively as the mass was abutting the iliac vessels and the right ureter. Intraoperatively, bilateral ureteric stent were inserted by the urology team. Exploratory laparotomy was performed, and the retroperitoneal mass was visible. Dissection was carried out circumferentially around this retroperitoneal mass until it was completely mobilized. A feeding lumber vessel was ligated with vascular ties (Figure 3), and complete excision of the mass was achieved.

Histological examination of the specimen grossly showed a large mass measuring 10.0 cm (superior to inferior) x 9.0 cm (medial to lateral) x 5.0 cm (anterior to posterior); it was tan-pink with a homogenous cut surface and soft in consistency. Histopathologically, the mass showed mature ganglioneuroma completely excised, and the International Neuroblastoma Pathology Classification (INPC) was Favorable histopathology.

The patient was doing well post-operatively, and the course was uneventful. She was discharged home after one week. The case was discussed with the tumor board for the next step in management and the decision was to follow up with annual surveillance.

During her recent follow-up to the clinic, one month postoperatively, she was doing great, her symptoms resolved, and she will be followed up annually with MRI.

## Discussion

Ganglioneuromas are difficult to diagnose as they are usually asymptomatic [6]. However, our patient presented with lower abdominal pain associated with menstruation, which could be related to the mass effect on the pelvic organs. The definitive diagnosis of ganglioneuromas is based on histopathology, and it appears under the microscope as a spindle cell tumor composed of neuritic processes, Schwann cells, and perineural cells, and shows numerous ganglion cells [3,5]. Imaging features of our case were similar to previous literature reports [2-6]. It was a homogeneous mass without calcification and had heterogeneous progressive enhancement on the post-contrast images on CT, while on MRI, it showed a high T2 signal and low T1 signal intensity with peripheral enhancement on the early images. The treatment of retroperitoneal mass is complete surgical excision [7]. Although resection can be made either via open or laparoscopic technique, open surgery is favored when operating on a tumor near or surrounding major blood vessels in the abdomen [8]. Some cases require preoperative or postoperative chemotherapy or radiotherapy if the mass is associated with ganglioneuroblastoma changes [5]. Interestingly, Ganglioneuroma tends to partially or entirely surround blood vessels without compromising the lumen in most cases, as seen in our case [9]. Ganglioneuroma has a low recurrence rate and a good prognosis, and in the majority of cases it does not require chemotherapy, radiation therapy, or other treatments following complete resection [5].

## Conclusions

Ganglioneuroma is a benign subtype of neuroblastic tumors that are usually found incidentally. Rarely, they may cause symptoms, as shown in our case report. A thorough assessment and investigation should be done to establish the diagnosis. Moreover, full work-up is also helpful for intraoperative planning as it usually presents in proximity to major vessels. Complete surgical removal is associated with good prognosis and low recurrence rate.
